# Porcelain Laminate Veneers and Zirconia Abutment: The Winning Duo for Esthetic Management of the Anterior Maxilla

**DOI:** 10.1155/crid/8352691

**Published:** 2026-02-23

**Authors:** Ines Azouzi, Obaid Garouachi, Ilhem Ben Othmen, Sarra Nasri, Med Bassem Khattèche

**Affiliations:** ^1^ Department of Oral Surgery of the Military Hospital of Tunis, University of Monastir, Tunis, Tunisia, um.rnu.tn; ^2^ Department of Fixed Prosthodontics of the Dental Clinic of Monastir, University of Monastir, Monastir, Tunisia, um.rnu.tn

**Keywords:** diastema, esthetic, implant supported crown, porcelain laminate veneers, zirconia abutment

## Abstract

Achieving optimal esthetics, function, and biocompatibility in such cases often necessitates the integration of all‐ceramic restorations and dental implants. Single‐tooth implant‐supported restorations, especially in the anterior region, remain a challenging yet preferred option for tooth replacement. The choice of abutment material, particularly zirconia over metal, is critical for ensuring favorable esthetic outcomes. For diastema closure, porcelain laminate veneers are commonly indicated, providing a conservative and effective solution for esthetic and functional concerns. This manuscript presents a clinical case illustrating the use of porcelain veneers and an implant supported prosthesis for the esthetic rehabilitation of the maxillary anterior region. The outcomes highlight the importance of adhering to clinical protocols to achieve optimal mechanical, biological, and esthetic results.

## 1. Introduction

In recent years, the pursuit of an esthetically pleasing smile has garnered significant attention, driven largely by the influence of celebrity culture and heightened public awareness. Consequently, the demand for esthetic dental treatments has become a daily challenge for dental professionals. The quest for the ideal smile has led to an increased focus on complex prosthetic restorations, particularly in cases involving missing incisors and multiple diastemas. Achieving optimal esthetics, function, and biocompatibility in such scenarios often necessitates the integration of various all‐ceramic restorations, dental implants, and their combinations [1].

The single‐tooth implant‐supported restoration has long been a preferred treatment option for replacing missing teeth, particularly in the anterior region. However, this procedure remains one of the most challenging aspects of contemporary dental rehabilitation. The choice of abutment material plays a critical role in the success of these restorations, especially in the esthetic zone. Metal abutments, while functional, can adversely affect the appearance of the peri‐implant gingival tissue and the final color of the restoration. In contrast, zirconia abutments are increasingly favored due to their ability to enhance the esthetic outcome. The inherent white color of zirconia eliminates the risk of darkened peri‐implant tissues and allows for a more natural integration with the all‐ceramic crown [2].

In cases involving diastema closure, porcelain laminate veneers (PLVs) are frequently employed to address both esthetic and functional concerns. Veneers, which require minimal enamel preparation, offer a conservative approach while achieving superior esthetic results. They are particularly indicated for correcting defects in tooth color, position, and form [3, 4].

This manuscript presents a clinical case detailing the closure of multiple spaces between maxillary anterior teeth using porcelain veneers, in conjunction with the replacement of a missing incisor via an implant‐supported prosthesis.

## 2. Case Report

A 32‐year‐old patient was referred to our prosthodontics department with the chief complaint of discrepancies in the size and form of the anterior teeth, as well as persistent spacing after orthodontic treatment. The patient′s medical history was noncontributory. A thorough anamnesis revealed that the patient had undergone 3 years of orthodontic treatment and had no deleterious oral habits.

**Figure 1:**
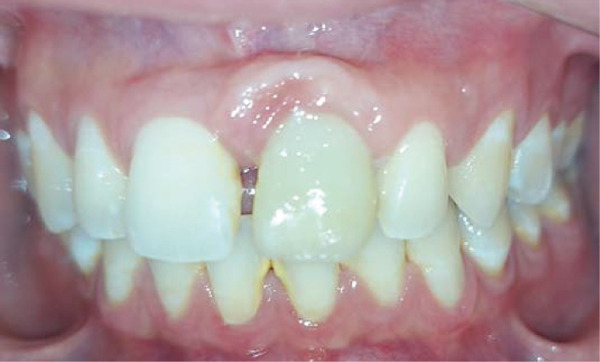
Initial situation: A frontal view reveals an arch length discrepancy, an unesthetic implant‐supported prosthesis on the left maxillary central incisor (#21), and a bulky gingival margin around Tooth #21, likely resulting from previous trauma.

**Figure 3:**
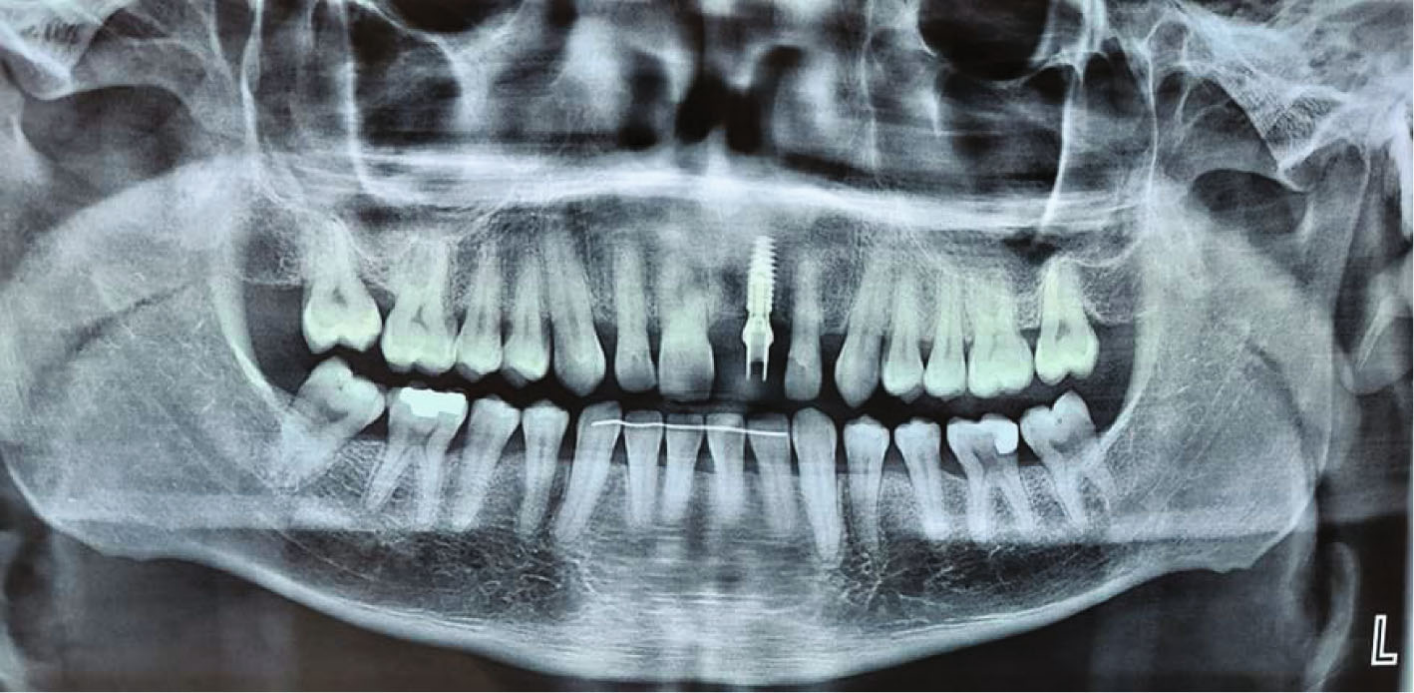
Panoramic radiographs: osseointegration of the implant at Site #21.

Intraoral examination revealed satisfactory oral hygiene, with an unesthetic implant‐supported prosthesis on the left maxillary central incisor (Tooth #21). The examination also noted diastema between the central incisors, as well as between the lateral incisors and canines on both sides of the upper arch. The gingiva surrounding the left central incisor exhibited a bulky appearance, which did not concern the patient, as he had a dental smile (Figures 1 and 2). Radiographic evaluation confirmed successful osseointegration of the implant, with no evidence of caries, bone loss, or root resorption (Figure 3).

Figure 2Initial situation: lateral view. (a) Left side and (b) right side.

**Figure:**
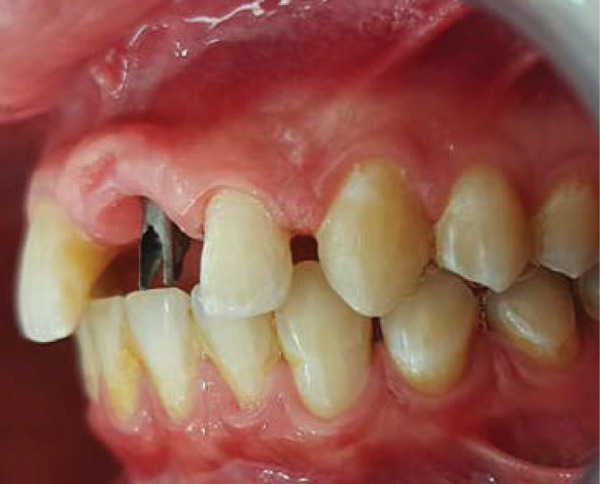
(a)

**Figure:**
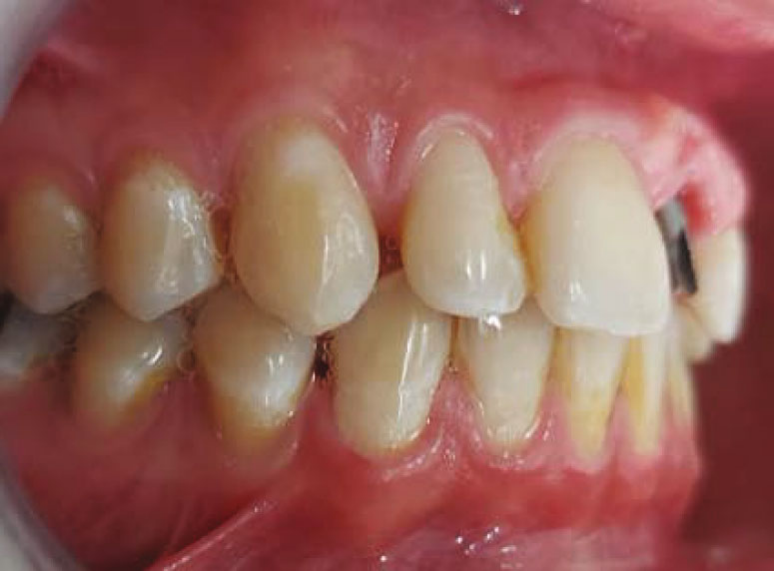
(b)

Several treatment options were discussed with the patient. He expressed a preference for a long‐lasting solution and opted for ceramic veneers to restore the form and close the diastema on Teeth #13, #12, #11, #22, and #23, owing to their superior optical properties, natural appearance, and durability. The patient declined the use of direct composite resin due to concerns about long‐term color stability.

The first challenge in the rehabilitation process was closing the diastema without creating disproportionate tooth widths in relation to their lengths while maintaining harmony with the adjacent teeth. The second challenge was addressing the poor esthetics of the discolored implant‐supported prosthesis on Tooth #21. To improve the outcome, the titanium abutment on the left central incisor was replaced with a zirconia abutment, fabricated using CAD/CAM technology.

To ensure that the proposed treatment would meet the patient′s expectations, diagnostic impressions were taken to create study models. The study templates were meticulously analyzed to determine the optimal shape and size of the restorations with the assistance of diagnostic wax‐ups.

The diagnostic wax‐up served as a blueprint for the subsequent interdisciplinary treatment and helped the patient visualize the final outcome of the treatment.

A preoperative mock‐up was then fabricated to simulate the final esthetic outcome for patient approval. This mock‐up, which reproduced the diagnostic wax‐up, allowed for the reevaluation of the patient′s function, tooth length, incisal profile, and finer details and helped the patient visualize the final treatment outcome.

Phonetic tests were performed, and the patient was able to pronounce “F,” “S,” and “V” sounds without disturbances. Additionally, the evaluation of the maxillary central incisor exposure with the lips at rest revealed an appropriate amount of visible teeth, confirming the treatment plan as outlined in the wax‐up. The mock‐up also facilitated a less invasive dental preparation, ensuring maximum enamel conservation. Depth cutters were utilized to precisely control the amount of tooth structure removed. The entire surface of the mock‐up was cut back until the demarcation lines were eliminated, indicating that the required depth had been achieved. The entire surfaces were then reduced using a round‐ended diamond bur at various angles, following the natural convexity of the tooth.

### 2.1. Dental Preparation (Figure 4)

Figure 4(a) Preparation on Teeth #11, #12, and #13. (b) Preparation on Teeth #22 and #23. On the diastema side, the gingival preparation should be located subgingivally so that the emergence profile of the restoration can be slightly over contoured. The resulting gentle push on the papillae will produce the desired triangular shape.

**Figure:**
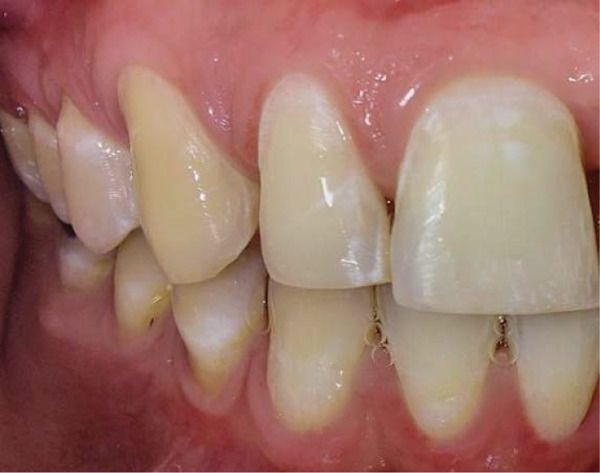
(a)

**Figure:**
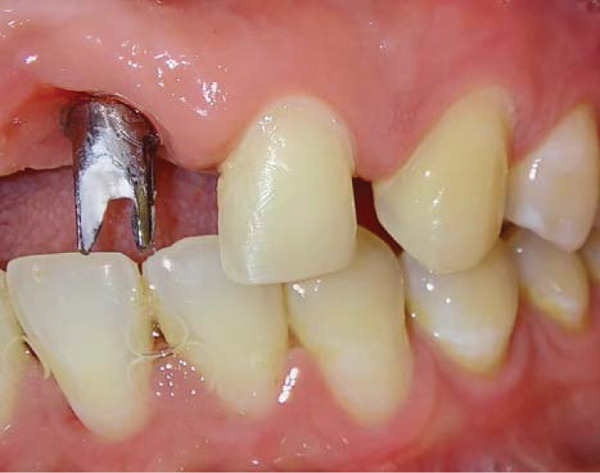
(b)

In the mock‐up‐driven technique, tooth preparation is performed on the mock‐up as if it were a natural tooth, resulting in a considerably less invasive approach. This method considers the final contour desired for the veneer, with diamond burs of preestablished depths used to achieve the necessary reduction. A cervical groove was created with a rounded diamond bur to outline the future cervical finish line. Three horizontal grooves were made with a depth marker bur on the labial surface. The reduction of the labial surface was then performed in three different planes (cervical, middle, and incisal thirds) to an enamel depth of 0.5–0.7 mm. Proximal preparation was extended beyond the contact area to avoid visibility of the margin and the appearance of a black triangle. Incisal edge reduction of 1.5 mm was performed, with a chamfer finish line maintained at the cervical region at the gingival margin level.

Due to the presence of a diastema, the preparation design was modified to feature a “slide” configuration, enabling the ceramist to create a natural‐looking contact area without the need for a lingual ledge. Depending on the porcelain thickness, the preparation may start subgingivally to provide adequate space for creating a natural clinical crown and facilitate the shaping of the gingival papilla.

Proximal preparation is divided into two key regions: the gingivo‐proximal and the proximal areas.

*Gingivo-proximal region*: As the interdental papilla is crucial for esthetic outcomes, it is essential to avoid penetrating the dentogingival complex or crossing the cementoenamel junction during proximal margin preparation. The bur should be angled at 60° relative to the tooth′s long axis, directed toward the palatal surface. The location of the gingival zenith point should be part of the treatment planning, as unchanged zenith points can make the teeth appear mesially tilted. Thus, the zenith points should be relocated through a periodontal procedure or by recontouring the gingival trough in provisional restorations.

*Proximal region*: To manage the diastema, it is important to prepare the proximal surfaces extensively, extending beyond the contact areas while maintaining a layer of enamel to support PLVs and ensure that the restoration–tooth interface remains concealed.

In some cases, slightly oversized veneers may be used in the gingival embrasure to allow the gingival papilla in the diastema to transition from a blunted to a more knife‐edged appearance. The path of insertion for the veneers should be free from undercuts, and sharp angles should be rounded during refinement.

### 2.2. Impression Procedure

A one‐phase impression was taken with putty and light body polyvinyl siloxane material and sent to the laboratory.

### 2.3. Temporization

Temporization of the tooth preparation was performed to maintain the patient′s appearance and contribute to the overall success of the restoration.

Provisional restorations involved spot‐etching the center of the buccal surface, followed by the application of a bonding resin and a direct light‐cure composite resin build‐up.

### 2.4. Computer‐Aided Design and Computer‐Aided Manufacturing

At the prosthetic laboratory, the impressions were poured, and the working, antagonist, and opposing models were scanned to record the patient′s occlusion. The veneers, crown, and customized abutment were virtually designed using the CEREC inLab CAD/CAM system (Sirona, Germany). The abutment was digitally constructed by integrating the implant‐specific connection base within the software. Following completion of the design, the digital files were transmitted to the milling center, where the abutment and restorations were fabricated with high precision and shade adaptation according to the clinical prescription.

### 2.5. Material Selection and Try‐in of Porcelain Veneers (Figures 5 and 6)

Figure 5(a) Labial and (b) occlusal views of the CAD/CAM design of the abutment.

**Figure:**
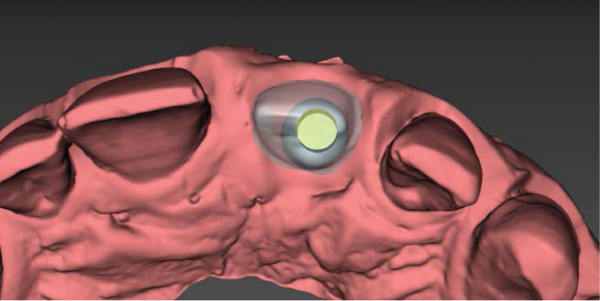
(a)

**Figure:**
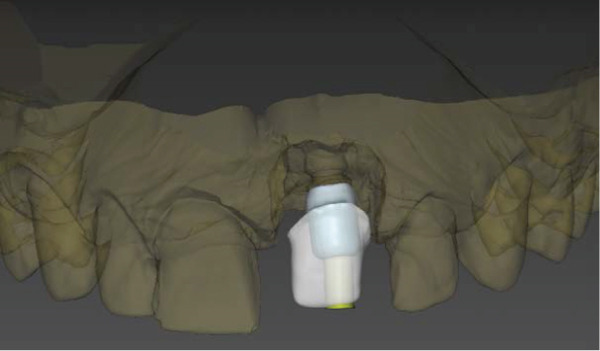
(b)

**Figure 6:**
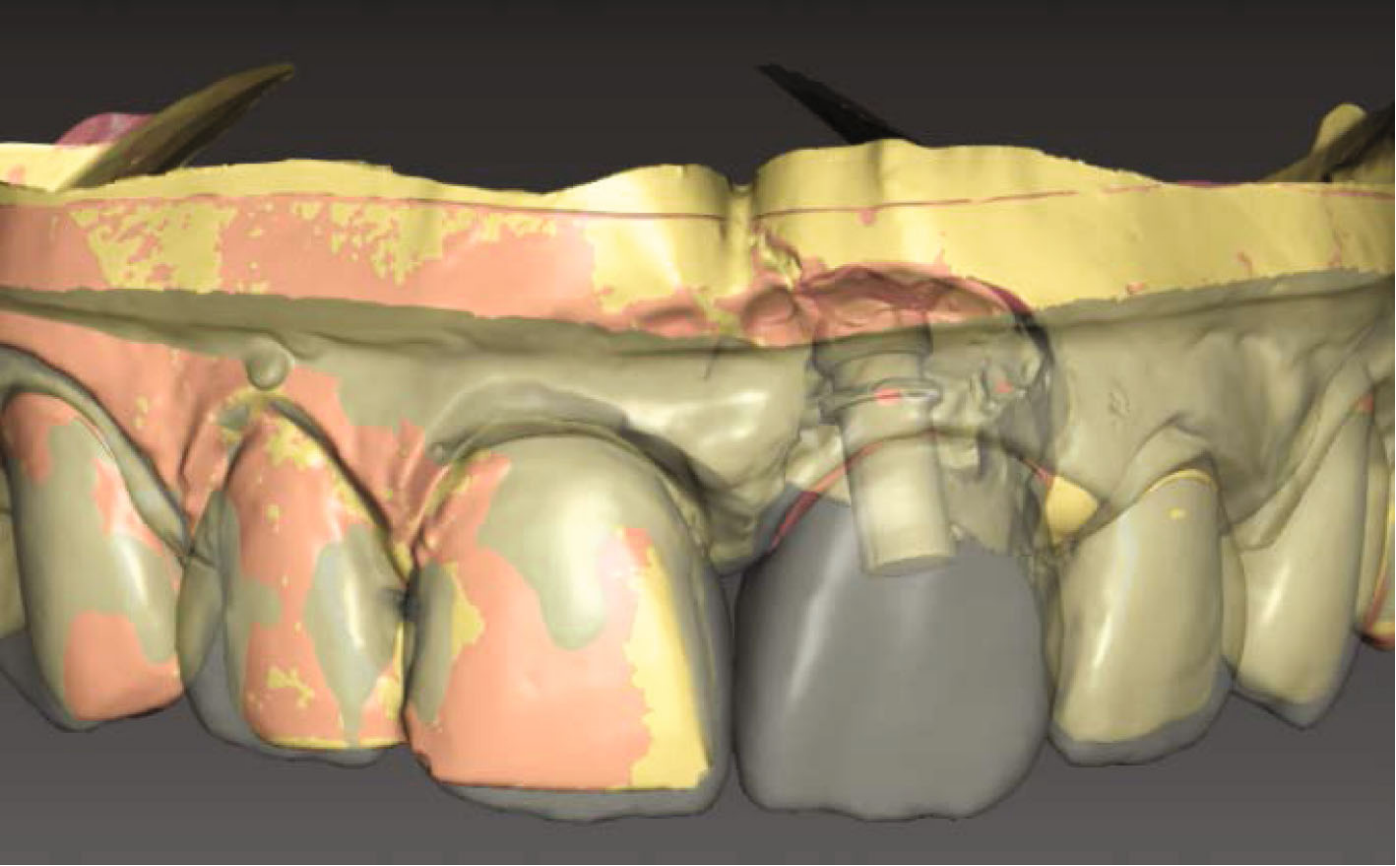
Labial view of the CAD/CAM design of the implant‐supported crown and veneers.

The veneers were fabricated from lithium disilicate–reinforced glass ceramics (IPS e.max Press, Ivoclar Vivadent) with a mean thickness of 0.7 mm, in accordance with the DSD and mock‐up planning specifications. During the subsequent visit, the veneers were tried in for evaluation of color, contour, fit, marginal adaptation, shape, size, symmetry, esthetic outcome, and harmony with the smile. The initial dry test was followed by a wet test using try‐in paste to simulate the effect of the resin cement on the final esthetic result. The patient approved the veneers.

### 2.6. Bonding of Porcelain Veneers and the Implant Supported full Coverage Crown (Figures 7, 8, 9, 10, 11, 12, 13, 14, 15, and 16)

**Figure 7:**
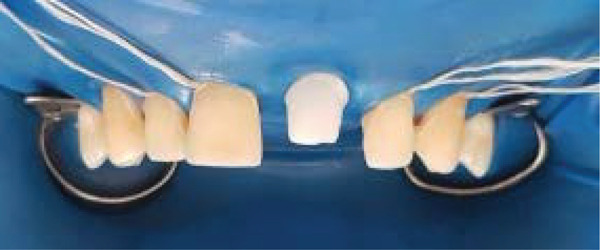
Rubber dam application.

As in any bonding procedure, absolute dental and gingiva isolation with rubber dam are a must (Figure 7).

**Figure 8:**
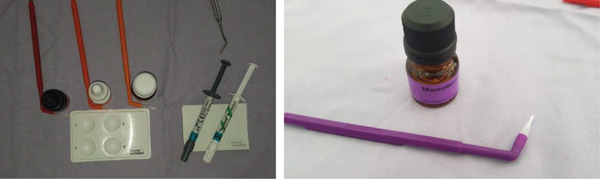
Bonding materials.

**Figure 9:**
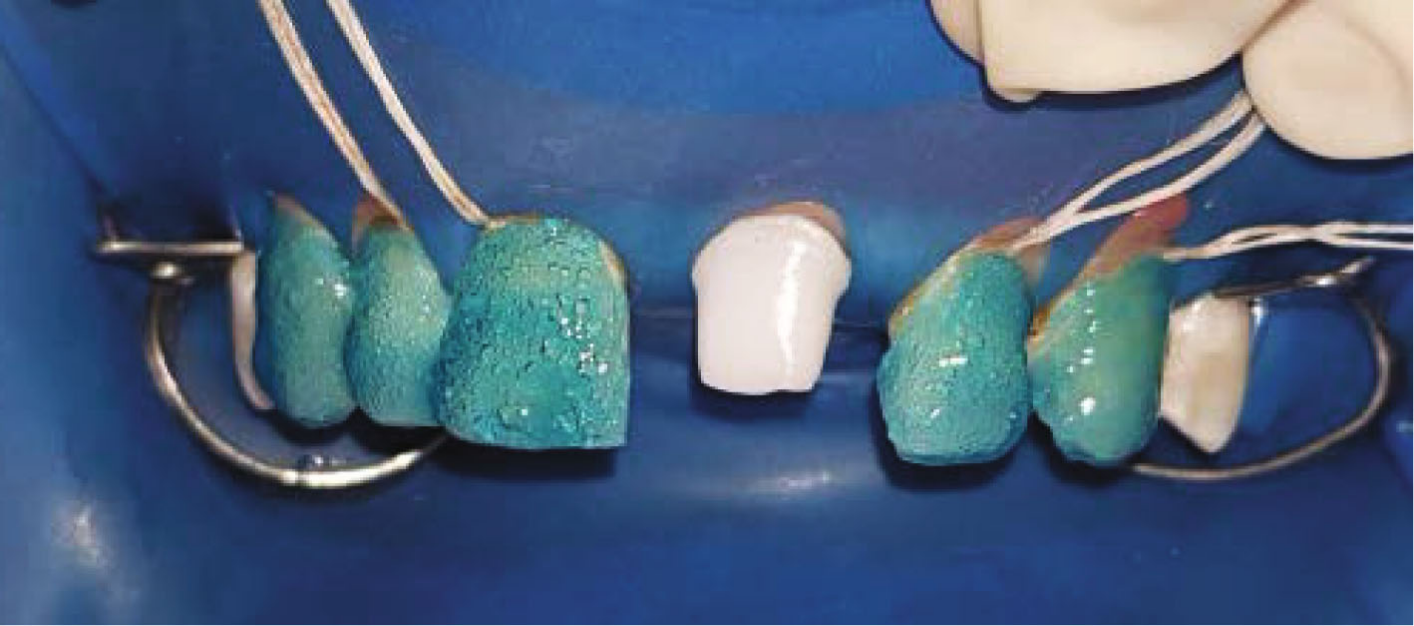
Orthophosphoric acid etching.

**Figure 10:**
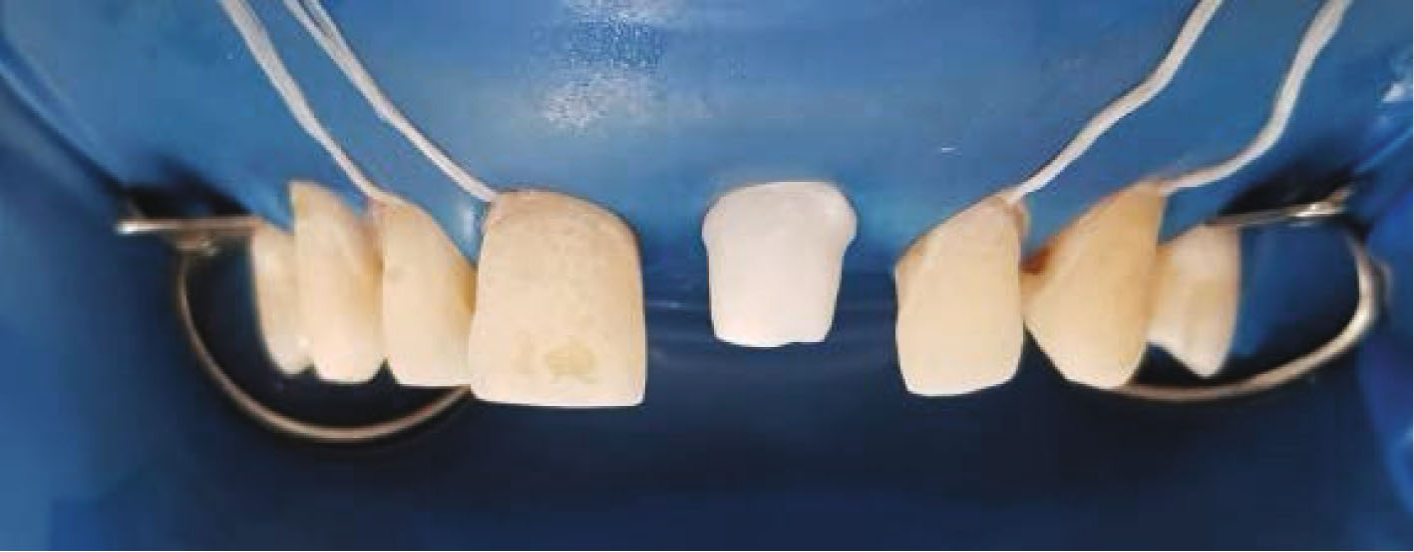
White chalky aspect.

**Figure 11:**
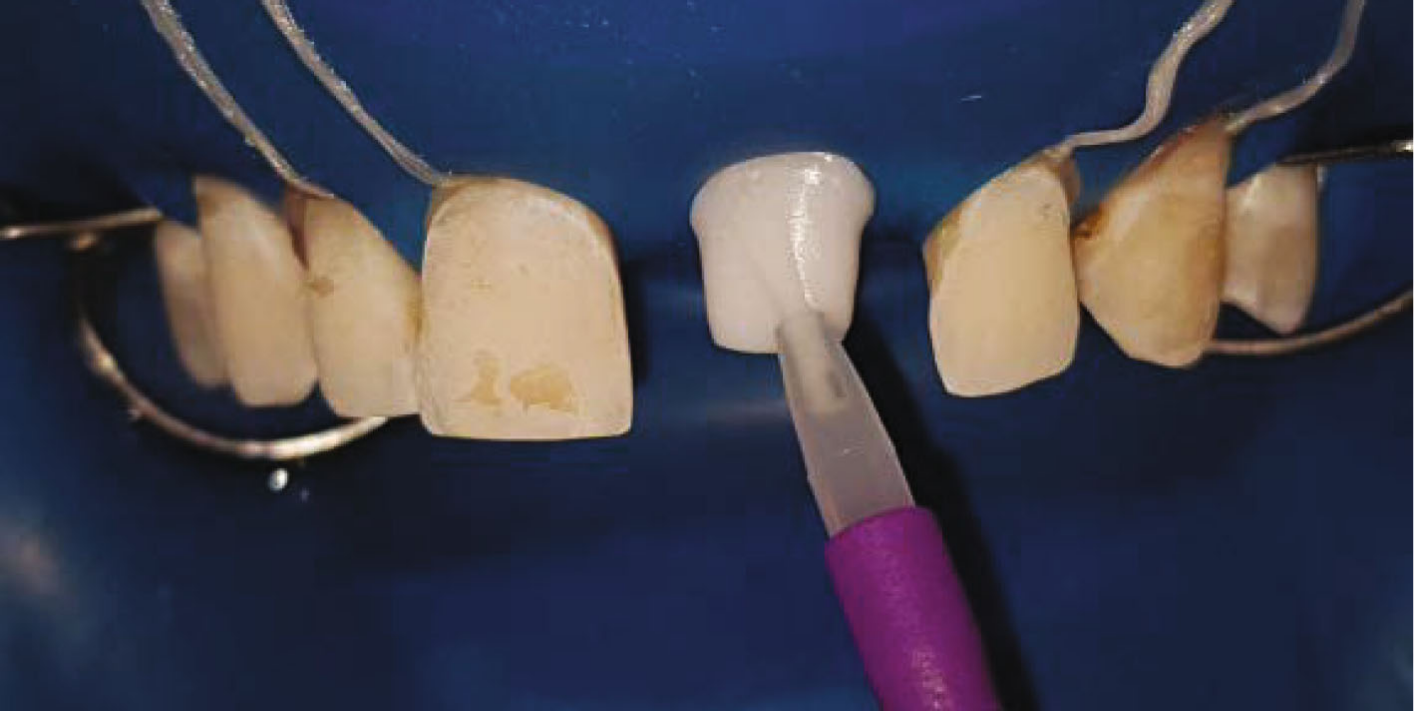
Application of silane on the abutment and the bonding agent on the teeth.

**Figure 12:**
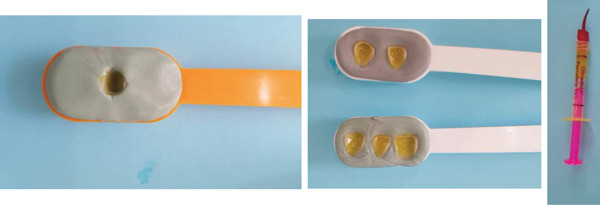
Preparation of the inner surface of the veneers as well as the implant supported full coverage crown. Prior to etching, the outer surface must be protected with silicon.

**Figure 13:**
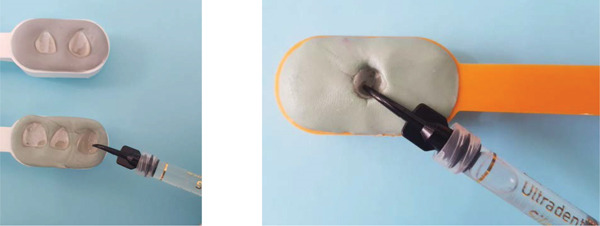
Silanization of the inner surface of the restorations. Once etching is complete, the surface is thoroughly rinsed and dried until a chalky white aspect is obtained.

**Figure 14:**
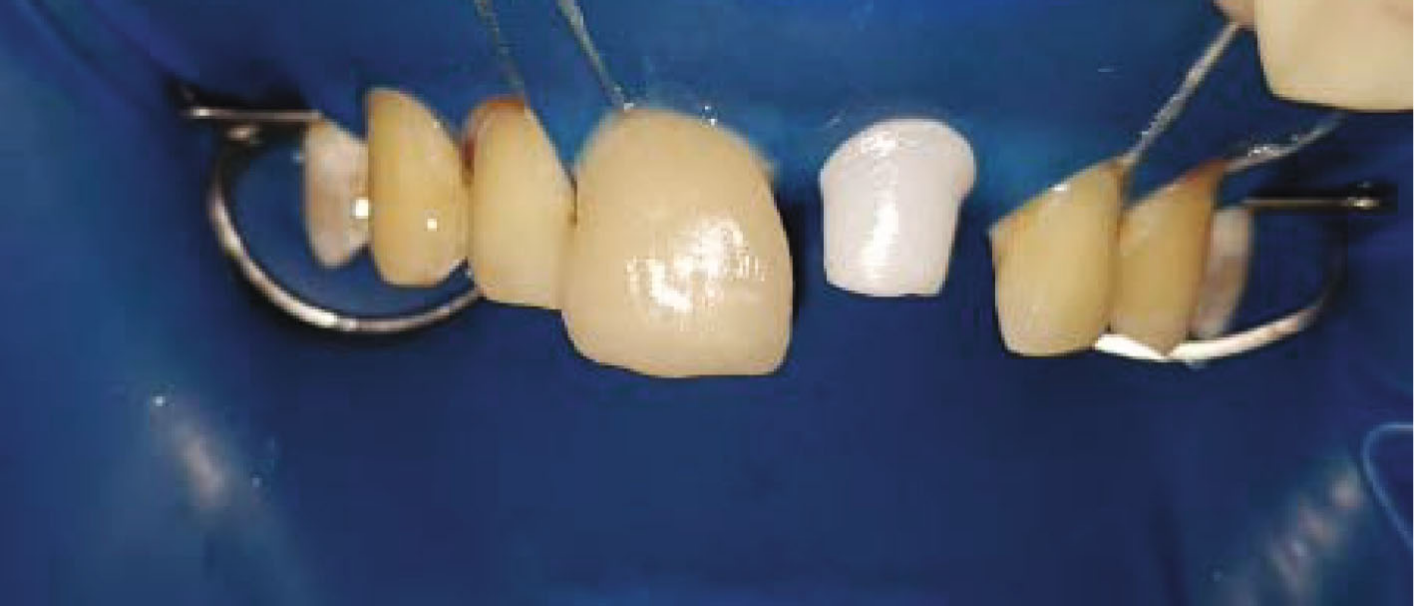
Luting of the laminate veneers with light‐curing resin cement.

**Figure 15:**
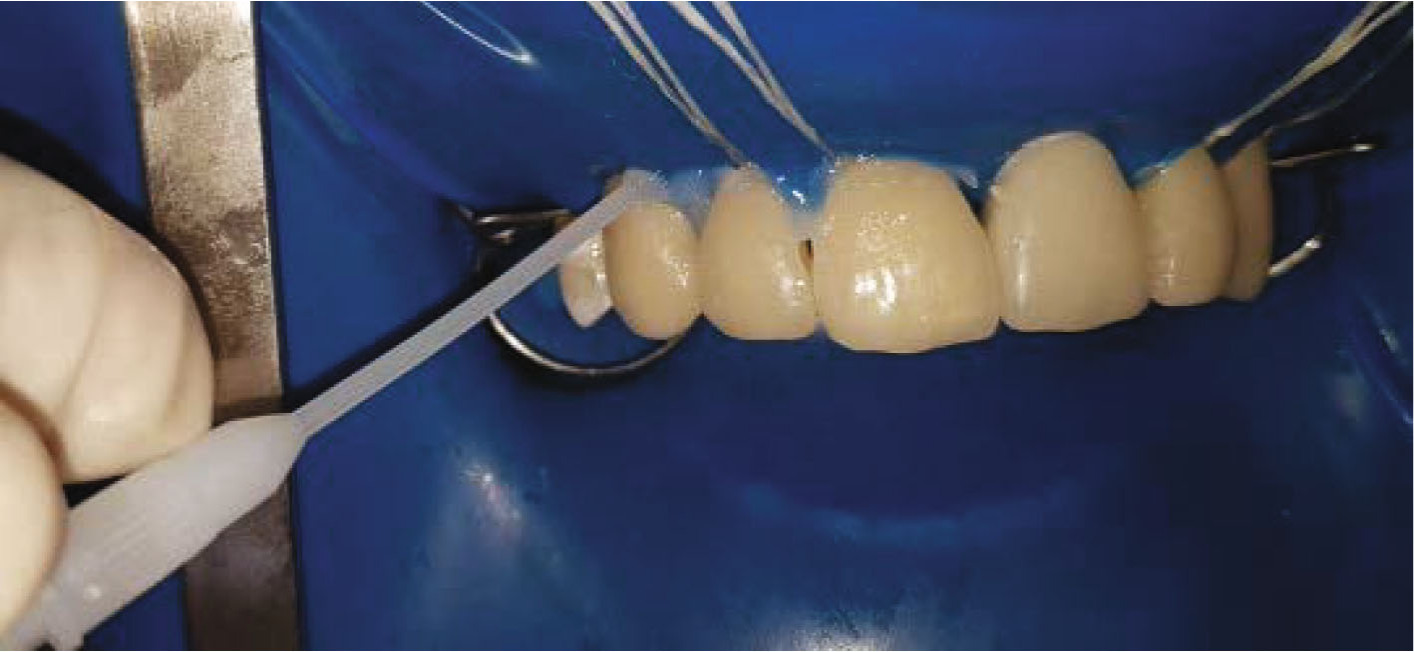
Application of glycerin gel on margins to eliminate oxygen inhibition layer before final curing.

**Figure 16:**
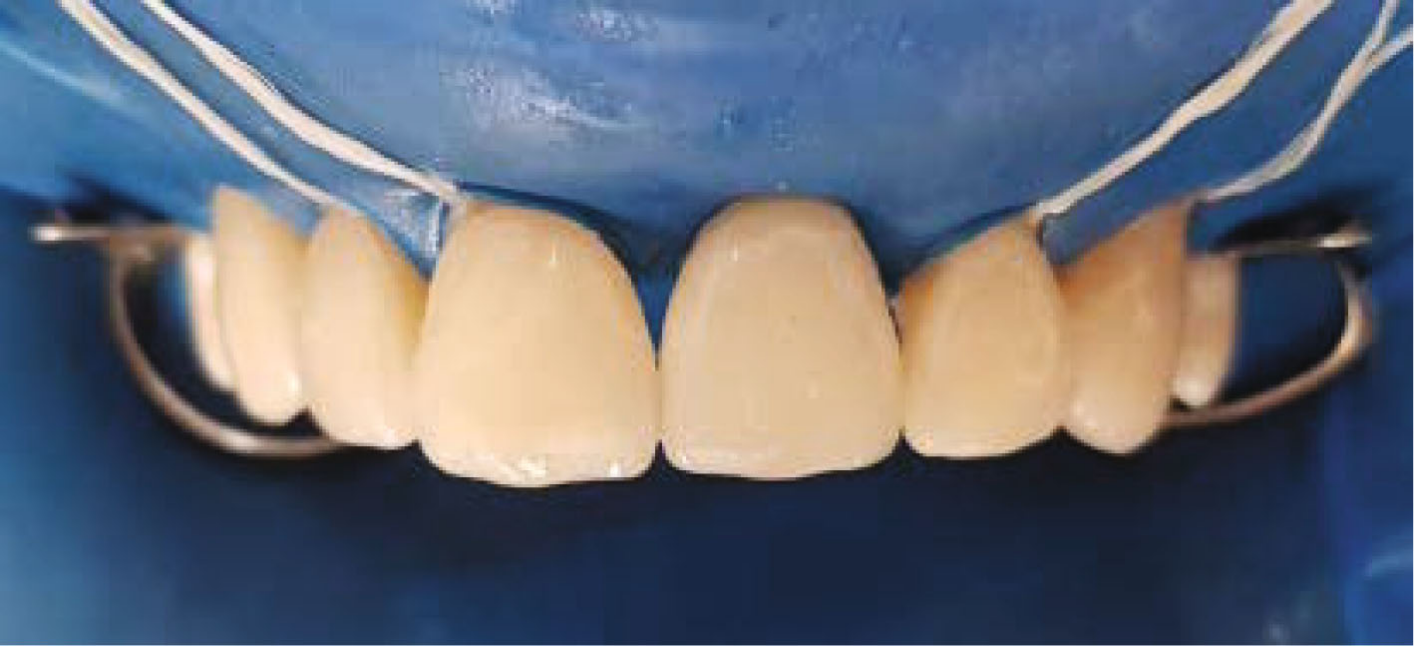
Intraoral frontal view after bonding of ceramic restorations.

The inner surface of each veneer was etched with 10% hydrofluoric acid for 20 s, rinsed thoroughly with water (Figure 12), dried until the appearance of white chalky aspect (Figure 10), and treated with a silane coupling agent to enhance bonding with the resin cement (Figure 13). The prepared teeth were etched with 35% phosphoric acid for 15 s (Figure 9), following the manufacturer′s recommendations, rinsed, and coated with a bonding agent, which was applied in a thin layer and light‐cured for 20 s (Figure 11). A translucent shade of luting cement was selected and applied to the inner surface of the PLVs (Figure 14).

Due to the delicate nature of porcelain veneers, careful handling was essential during both the try‐in and cementation steps to avoid fracture. The veneers were seated with gentle finger pressure on the buccal surfaces of the teeth, as excessive pressure could result in fracture, particularly when using high‐viscosity resin cement. Upon completion of the cementation (Figure 8), occlusion was checked in both centric relation and during protrusive mandibular excursions. Any contacts on the lingual aspects of the newly bonded veneers were removed and polished.

The zirconia abutment was seated, and the screw was torqued (35 Ncm). A sterilized polytetrafluoroethylene (PTFE) tape pellet was placed into the screw access channel over the screw head. Following this, the supragingival porcelain surfaces, which had been previously etched and treated with silane, were coated with adhesive resin. A resin‐based luting agent was then applied, and the crown was seated and stabilized for light‐curing (Figure 16).

**Figure 17:**
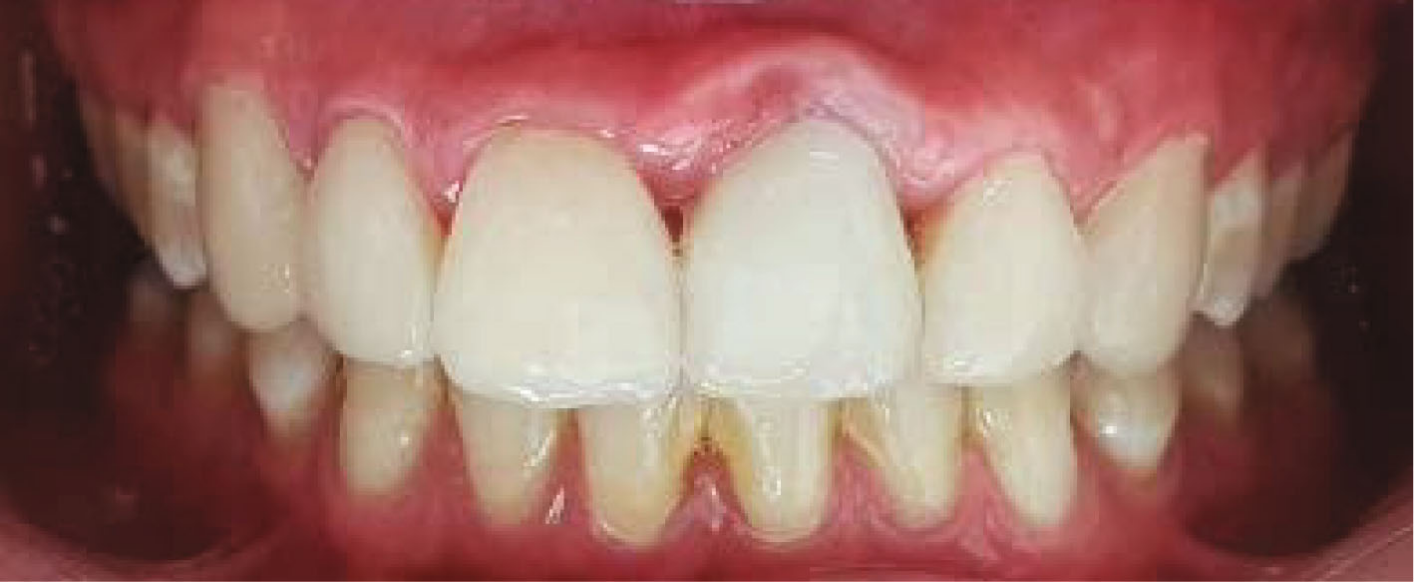
Final result after the esthetic rehabilitation.

**Figure 18:**
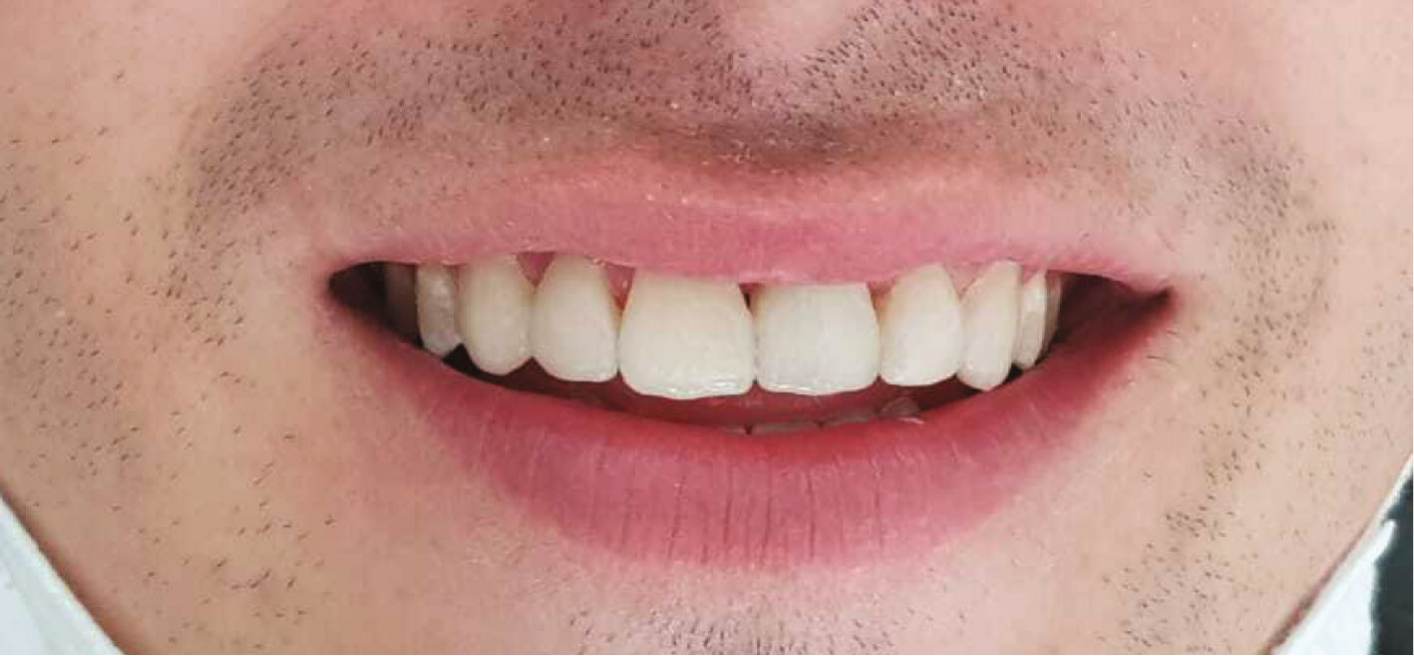
Final appearance of the patient′s smile.

The final result demonstrated excellent integration of the restorations with the gingival tissue, achieving a seamless, mimetic effect with the natural dentition (Figures 17 and 18). During recall appointments, checks were performed for sensitivity, infiltration, or fractures on both the teeth and the PLVs. The patient was informed about the importance of proper aftercare and advised to avoid biting on hard objects to prevent restoration fractures, as well as to maintain meticulous oral hygiene using interdental brushes and floss.

The patient was advised to floss carefully after toothbrushing and to attend regular follow‐up dental visits to monitor the restoration′s condition. Additionally, he was instructed to avoid chewing hard substances, such as candies, and to prevent thermal shock by avoiding the rapid consumption of hot drinks followed by cold ones.

The success of prosthetic rehabilitation is largely dependent on the careful selection of an optimal treatment plan and restoration type, along with a thorough understanding of the techniques and materials used. Collaboration with a skilled dental technician is also critical to achieving favorable outcomes.

While the esthetic results achieved with zirconia abutments are highly promising, further clinical studies are necessary to validate their long‐term efficacy.

## 3. Discussion

A diastema, or maxillary anterior spacing, represents an esthetic concern with a higher prevalence in the maxilla compared to the mandible, and it typically arises from a multifactorial etiology [5]. The presence of these spaces can detract from an otherwise harmonious smile, drawing the observer′s attention away from the overall dental composition and focusing instead on the diastema [6]. Additionally, diastema can contribute to phonetic impairments [7].

The initial approach to treating diastema involves identifying the underlying cause. Diastemas can be attributed to several factors: hereditary factors, including congenitally missing teeth, discrepancies between tooth and jaw size, supernumerary teeth, and abnormal frenum attachments, and developmental issues, such as oral habits, periodontal disease, tooth loss, and posterior bite collapse [8, 9].

Several treatment modalities can be considered for addressing diastema. Orthodontics is often proposed as a treatment modality, offering significant esthetic improvements and being generally well‐accepted by patients. However, orthodontic treatment alone may be insufficient to address issues related to excessive spacing, particularly in cases involving tooth‐size discrepancies. Dentoalveolar discrepancies, often cited as a common cause of anterior diastema in adults, are characterized by an imbalance in the size and shape of the teeth relative to the dental arches, which can prevent proper occlusal contact and result in single or multiple diastema [10]. These discrepancies are typically due to disharmony between the dental arch size and tooth width or the presence of bone defects [11].

In situations where orthodontic intervention cannot fully correct the spacing issues, restorative, prosthodontics, or even periodontal procedures may be necessary to achieve closure of the diastema. The choice of treatment should be determined after thorough documentation and discussion with the patient, taking into account their specific esthetic concerns and expectations.

The initial challenge in treating diastema begins with a detailed anamnesis, as understanding the patient′s dissatisfaction with their appearance and their expectations is crucial for determining feasible treatment options and recognizing any limitations [7].

Study models and wax‐ups are essential tools for assessing clinical conditions, evaluating restoration forms, and planning occlusal and esthetic designs [12]. Accurate measurements derived from the study model, wax‐up, and photographs are indispensable during treatment planning, as they help in determining the width of the diastema and identifying esthetic discrepancies. The mock‐up, created with bisacrylic resin, plays a significant role in allowing both the dentist and the patient to select an esthetically acceptable shade, choose the appropriate material, and refine the shape of the teeth [7, 13].

When employing a restorative approach to close diastema, achieving correct tooth proportions despite the mesiodistal enlargement of the teeth adjacent to the gap is a key challenge. For gaps of 1 mm or less, where the teeth are near ideal proportions, only minimal additions may be required, minimizing any negative impact on tooth proportions. Tooth characterization techniques such as adjusting the line angles or rounding the distal–incisal corners can be used to create the illusion of narrower teeth. If maintaining proper length‐to‐width ratios necessitates tooth lengthening, this can be achieved apically through periodontal procedures or incisally through restorative techniques. However, when diastema closure results in short clinical crowns, a more aggressive restorative approach may be required, potentially involving multiple teeth [14].

Phonetic tests must be performed to ensure correct pronunciation without disturbances to the phonemes “F,” “S,” and “V” [10]. If speech alterations occur, patients should be informed that adaptation typically happens within a few days, with reading aloud serving as a helpful exercise for restoring previous speech patterns [7, 10].

Direct composite resin restorations and direct composite veneers are conservative options for diastema closure, often resulting in esthetically pleasing outcomes. These techniques enhance both esthetics and function while typically requiring minimal or no tooth preparation. Modern composite materials are not only esthetically appealing, durable, and affordable, but they also have a well‐documented long‐term adhesion to enamel. Advances in the physical and chemical properties of composites have further optimized color stability and improved wear resistance [6].

Additionally, direct composites offer benefits such as ease of intraoral repair, the ability to sculpt the restoration chairside, reduced costs, and the ability to complete the procedure in a single visit without laboratory fees [15].

However, composite resin has some drawbacks, including a tendency for discoloration and degradation over time [16]. Furthermore, the surface texture of composite veneers may not appear as natural as that of porcelain veneers. Many clinicians also find it more challenging to create precise anatomical details directly in the mouth compared to a technician working in a laboratory setting. Failure to accurately reproduce anatomic nuances may lead to an unesthetic, V‐shaped diastema closure [7, 15, 17].

In contrast, indirect techniques provide superior esthetics, color stability, and better surface texture. Porcelain veneers, for instance, exhibit a glazed surface similar to natural teeth, which promotes periodontal health by resisting plaque adherence and eliciting a favorable tissue response [7, 17, 18]. Additionally, indirect techniques prevent residual polymerization and polymerization shrinkage since these processes occur outside the mouth, ensuring more predictable patient outcomes.

Currently, PLVs have become a preferred alternative to composite restorations for patients who are well‐informed, value esthetic standards, and seek minimally invasive treatments. The widespread acceptance of PLVs is attributed to their excellent esthetic results, minimal biological cost, high biocompatibility, long‐term color stability, minimally invasive preparation, mechanical properties similar to human enamel when bonded, and superior abrasion resistance [19].

Due to their optical properties, PLVs are highly capable of replicating the natural characteristics of teeth. Ceramics exhibit natural fluorescence and can absorb, reflect, and transmit light in a manner similar to natural tooth structure [20]. Furthermore, several studies have demonstrated that ceramic veneers have greater longevity than direct composite veneers in terms of success and survival rates [21]. For instance, according to Arif et al., PLVs showed an estimated survival probability of 0.976 over 7 years and 0.882 over 14 years, with a high survival rate of 98% and a low failure rate of 4.38% [22].

J. Calamia and C. Calamia outlined several key factors that contribute to the long‐term success of porcelain veneers, potentially extending their lifespan to up to 25 years. These factors include thorough treatment planning, preparation terminating in enamel, appropriate selection of ceramic material, and proper cementation techniques [23]. Preserving enamel, particularly at the margins, is crucial for the success of laminate veneers, as dentin exposure can adversely affect adhesive strength and reduce the longevity of the restoration [24]. Öztürk and Bolay demonstrated that PLVs exhibit high survival rates when bonded exclusively to enamel or to enamel with minimal dentin exposure. However, extensive dentin exposure should be avoided during preparation [25].

Zhu et al. also emphasized that bonding to 100% enamel is the most reliable approach for achieving optimal results. When dentin exposure is unavoidable, enamel should be preserved as much as possible to maintain good bonding. Zhu et al. further noted that at least 40% of the bonding surface should consist of preserved enamel to ensure adequate bond strength [26].

The mock‐up driven preparation technique is designed to ensure minimally invasive and guided preparation while preserving tooth structure. This technique also takes into account the final contour desired for the veneer [27, 28]. According to Gurel et al., the mock‐up driven technique resulted in 80.5% of tooth preparations being confined to dental enamel, leading to more predictable outcomes [29].

Several studies have highlighted the importance of immediate dentin sealing after the exposure of dentinal tubules during preparation. This technique is crucial for enhancing adhesion, protecting the pulp, and preventing bacterial infiltration and postoperative sensitivity [30, 31].

For successful management of the gingival architecture, the cervical contour can be prosthetically adjusted by repositioning the zenith point during preparation. For a gradual and natural closure of the diastema, intrasulcular placement of the cervical proximal margins is recommended, along with the strategic positioning of the contact point to improve the emergence profile [32].

Ceramic materials used for porcelain veneers encompass various types, including feldspathic porcelain, feldspathic porcelain reinforced with leucite, lithium disilicate, and lithium disilicate reinforced with zirconia [33]. In this clinical case, the veneers are fabricated using lithium disilicate–reinforced glass ceramic, which offers several advantageous properties, such as high strength, excellent marginal integrity, and superior esthetics. This material also provides reliable bonding due to its etchability. It is frequently chosen for their favorable optical properties, including various levels of translucency and opacity Moreover, this ceramic demonstrates slower crack propagation and enhanced fracture resistance [34]. According to a systematic review by Sailer et al., 12 studies reported that lithium disilicate reinforced glass ceramics had an estimated 5‐year survival rate of 96.6%, which is comparable to the survival rate of metal–ceramic restorations [35].

The selection of an adhesive for veneers is a critical decision that requires careful consideration by the clinician. With the wide variety of bonding systems available, choosing the appropriate system has become increasingly challenging. Regardless of the chosen system, it is crucial to apply the bonding protocol rigorously and adhere to the manufacturer′s guidelines. Three‐step etch‐and‐rinse adhesives systems remain the most effective and exhibit the least sensitivity to variations in application techniques. These adhesive systems are also associated with more durable bonding over extended periods [36]. For porcelain veneers, the use of photo‐cured resin cement is recommended because it maintains its color stability over time. In contrast, self‐polymerized and dual‐polymerized resin cements are prone to discoloration, potentially leading to undesirable esthetic changes in the veneers [37].

A critical factor in the success of PLVs is the precise occlusal adjustment following cementation. Proper adjustment ensures that minimal compressive and shear forces are exerted on the veneers, which is essential for their long‐term durability and longevity [38].

A key challenge in implant dentistry is achieving a gingival margin that mimics the appearance of the cementoenamel junction of a natural tooth. This is especially difficult with titanium implants fixtures and abutments, as their color and reflectivity can cause the metal to show through the gum tissue, leading to a grayish appearance. Discoloration of the gums can reduce patient satisfaction [39–41]. This problem has been around since porcelain‐fused‐to‐metal restorations were introduced and remains an issue with metal implants [42–44].

The thickness of the gum tissue plays a significant role in hiding or revealing this discoloration, which is more noticeable when the gums are thin and transparent. Additionally, nonprecious alloys can contribute to tissue discoloration due to corrosion [45]. Recently, zirconia has emerged as a material for crown infrastructure and implant abutments [46]. The literature highlights several advantages of ceramic abutments over metal abutments. These advantages include reduced mucosal discoloration, decreased bacterial adhesion, minimal or absent cytotoxicity, and comparable mucosal attachment to zirconia and titanium [47].

Degidi et al. performed a human histologic study to evaluate the peri‐implant soft tissue response to titanium and zirconium oxide healing caps. The study found that the peri‐implant soft tissues adjacent to titanium healing caps exhibited a higher incidence of inflammatory infiltrate compared to those around zirconium oxide healing caps [48]. This phenomenon can be attributed to the fact that ceramic have a smoother surface finish and are more easily polished than metal, significantly reducing bacterial colonization and adhesion [49]. Additionally, ceramic demonstrate resistance to corrosion, which promotes superior epithelial cell growth and further inhibits bacterial adhesion.

However, a notable limitation of ceramic materials is their mechanical properties, as they exhibit brittleness, which results in lower resistance to tensile forces [47, 50–53]. Numerous studies have demonstrated that zirconia can meet and even exceed known human occlusal forces. For instance, one study analyzing anterior zirconia, titanium, and aluminous porcelain test abutments after artificial aging found that the median fracture resistance of zirconia abutments was 443.6 N, which is near the upper range of human occlusal forces [54–58].

This finding indicates that zirconia is capable of withstanding the physiological occlusal forces encountered in the anterior region. Another strength test involving preaged zirconia crowns and implant abutments showed that zirconia fractures at a load high enough to withstand maximum human occlusal forces [59]. After processing, zirconia abutments exhibit a white color, and when combined with an all‐ceramic crown, this color does not show through the overlying tissues, thus overcoming the esthetic limitations associated with metallic components.

Bonding to zirconia presents a significant challenge due to the material′s inherent chemical stability and lack of a substantial glass matrix. These characteristics prevent the formation of the micromechanical undercuts typically generated by acid etching in other ceramic materials. Consequently, silane treatments, typically effective on silicate ceramics, are less beneficial for zirconia. Various mechanical and chemical surface treatments have been proposed to improve zirconia bonding efficacy, aiming to expand its utility in contemporary dental practice. The APC (air particle abrasion, zirconia primer, and adhesive composite resin) zirconia‐bonding concept, built upon extensive research, offers a practical approach to achieving strong and enduring bonds with high‐strength ceramics. Surface roughening of zirconia ceramics is commonly achieved through airborne particle abrasion technology [60, 61], which enhances the ceramic′s surface area and introduces pronounced asperities that improve bonding potential [62, 63]. The bonding efficacy largely depends on the ceramic′s surface texture, the resin cement′s wettability, and its chemical properties, including polymerization mechanisms. Notably, a study found that the shear bond strength of MDP‐containing resin cements to zirconia ceramics significantly improved with the application of a zirconia primer, whether the ceramic was polished or blasted. Additionally, effective moisture control is crucial for reliable adhesive bonding, especially with supragingival margins that facilitate easier moisture management compared to subgingival margins, where sulcular fluid can undermine the bonding integrity.

Despite the advantages of zirconia abutments, the interface between the zirconia and the titanium implant may be susceptible to accelerated wear under occlusal forces due to their material dissimilarities. Such wear can precipitate several complications including discoloration of the peri‐implant mucosa and loosening of abutment screws and fractures of the ceramic abutments and crowns [64, 65]. Compared to counterparts that utilize a titanium base, these abutments exhibit notably lower fracture resistance [66].

Therefore, scheduled maintenance visits are essential to ensure early detection of potential complications, allowing for prompt intervention and minimizing the risk of long‐term adverse outcomes. These routine evaluations play a critical role in maintaining the stability and functionality of the treatment.

## 4. Conclusion

Ceramic veneers are a highly effective option for closing diastemas and should be considered a primary treatment modality due to their ability to provide optimal esthetic results. Similarly, single‐tooth implant‐supported restorations are a preferred method for replacing missing teeth in the anterior region, particularly when zirconia abutments are used. Zirconia abutments enhance esthetics by preventing tissue discoloration and integrating seamlessly with all‐ceramic crowns. In both cases, strict adherence to clinical protocols is crucial for optimizing mechanical, biological, and esthetic outcomes, ensuring the success of the treatment.

## Conflicts of Interest

The authors declare no conflicts of interest.

## Funding

No funding was received for this manuscript.

## Data Availability

The data that support the findings of this study are available from the corresponding author upon reasonable request.
